# Structural basis for the Nanos-mediated recruitment of the CCR4–NOT complex and translational repression

**DOI:** 10.1101/gad.237289.113

**Published:** 2014-04-15

**Authors:** Dipankar Bhandari, Tobias Raisch, Oliver Weichenrieder, Stefanie Jonas, Elisa Izaurralde

**Affiliations:** Department of Biochemistry, Max Planck Institute for Developmental Biology, 72076 Tübingen, Germany

**Keywords:** deadenylation, decapping, mRNA decay, SLiM, translational repression

## Abstract

The RNA-binding proteins of the Nanos family play an essential role in germ cell development and survival in a wide range of metazoan species. They function by suppressing the expression of target mRNAs through the recruitment of effector complexes, which include the CCR4–NOT deadenylase complex. Here, we show that the three human Nanos paralogs (Nanos1–3) interact with the CNOT1 C-terminal domain and determine the structural basis for the specific molecular recognition. Nanos1–3 bind CNOT1 through a short CNOT1-interacting motif (NIM) that is conserved in all vertebrates and some invertebrate species. The crystal structure of the human Nanos1 NIM peptide bound to CNOT1 reveals that the peptide opens a conserved hydrophobic pocket on the CNOT1 surface by inserting conserved aromatic residues. The substitutions of these aromatic residues in the Nanos1–3 NIMs abolish binding to CNOT1 and abrogate the ability of the proteins to repress translation. Our findings provide the structural basis for the recruitment of the CCR4–NOT complex by vertebrate Nanos, indicate that the NIMs are the major determinants of the translational repression mediated by Nanos, and identify the CCR4–NOT complex as the main effector complex for Nanos function.

## Abstract (precis)

Nanos family RNA-binding proteins play essential roles in metazoan germ cell fate and survival, yet the underlying mechanism has not been elucidated. Bhandari et al. now provide the structural basis for CCR4–NOT complex recruitment by vertebrate Nanos. The authors show that three human Nanos paralogs interact with the CNOT1 C-terminal domain through a short, conserved CNOT1-interacting motif (NIM). This work indicates that NIMs are the major determinants of the translational repression mediated by Nanos and identifies the CCR4–NOT complex as the main effector for Nanos function.

The spatial and temporal regulation of mRNA expression plays an essential role during embryonic development. This regulation is mediated by sequence-specific RNA-binding proteins that inhibit the expression of their target mRNAs through the recruitment of effector complexes (Lasko 2011; Barckmann and Simonelig 2013). Nanos is a conserved post-transcriptional mRNA regulator that was first identified as being required for posterior pattern formation in the *Drosophila melanogaster* embryo (Lehmann and Nüsslein-Volhard 1991). In addition to embryonic patterning, Nanos plays an essential role in embryonic germline development, germline stem cell maintenance, and neuronal homeostasis in a wide range of metazoa (Jaruzelska et al. 2003; Tsuda et al. 2003; Baines 2005; Lai and King 2013).

Although some organisms, such as *D. melanogaster* and other insects, contain only one Nanos protein, two paralogs were identified in Hydra, and three were found in *Caenorhabditis elegans* and in most vertebrates (Subramaniam and Seydoux 1999; Mochizuki et al. 2000; Köprunner et al. 2001; Jaruzelska et al. 2003; Tsuda et al. 2003). These paralogs perform distinct but also partially redundant functions and display specific expression patterns (Mochizuki et al. 2000; Haraguchi et al. 2003; Jaruzelska et al. 2003; Kusz et al. 2009; Suzuki et al. 2007; Julaton and Reijo Pera 2011). For example, in mice, Nanos1 is expressed in oocytes, the adult brain, and testes, whereas Nanos3 is detected in primordial germ cells (PGCs), and Nanos2 is present in only male PGCs. Although no defects were found upon the loss of Nanos1 (Haraguchi et al. 2003), the knockout of Nanos2 or Nanos3 leads to the loss of germ cells in males or both sexes, respectively (Tsuda et al. 2003; Sada et al. 2009). The role of these proteins in germ cell maintenance and survival appears to be conserved in humans because all three Nanos paralogs were found to be expressed in the gonads (Jaruzelska et al. 2003; Kusz et al. 2009; Julaton and Reijo Pera 2011), and mutations in Nanos3 have been linked to premature ovarian insufficiency (Wu et al. 2013).

Nanos proteins consist of a conserved CCHC-type zinc finger domain and divergent N-terminal and C-terminal extensions of variable lengths and low sequence complexity, which are predicted to be unstructured (Fig. 1A). The zinc finger domain is essential for Nanos function (Curtis et al. 1997; Arrizabalaga and Lehmann 1999; Asaoka-Taguchi et al. 1999) because it mediates RNA binding and binding to Pumilio, a conserved Nanos partner that confers mRNA target specificity (Asaoka-Taguchi et al. 1999; Sonoda and Wharton 1999; Jaruzelska et al. 2003). The unstructured termini of Nanos proteins are required for their interaction with effector complexes (Arrizabalaga and Lehmann 1999; Verrotti and Wharton 2000; Ginter-Matuszewska et al. 2011). Although the Nanos partners vary depending on the organism and mRNA target, a common emerging partner is the CCR4–NOT deadenylase complex, which interacts with the N-terminal region of Nanos proteins in diverse organisms (Kadyrova et al. 2007; Suzuki et al. 2010, 2012; Joly et al. 2013).

**Figure 1.**
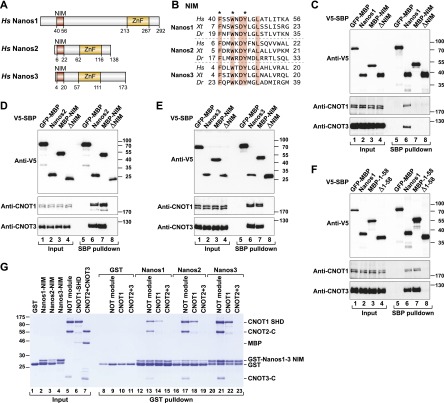
Human Nanos1–3 interact with the CNOT1 SHD directly through conserved NIMs. (*A*) The domain organization of human Nanos1–3. Nanos proteins consist of a conserved C-terminal CCHC-type zinc finger domain (ZnF; orange) and variable N-terminal and C-terminal extensions (gray). The NIMs are shown in red. The numbers *below* the protein outlines indicate the residues at the domain/motif boundaries. (*B*) Sequence alignment of vertebrate NIMs. The residues conserved in all of the aligned vertebrate sequences are shown with a salmon background. The asterisks indicate the residues that were mutated in this study. The species abbreviations are as follows: *Hs* (*Homo sapiens*), *Xt* (*Xenopus tropicalis*), and *Dr* (*Danio rerio*). (*C–F*) Interaction of V5-SBP-tagged Nanos1–3 (full length or the indicated mutants) with endogenous CNOT1 and CNOT3 in HEK293T cells. A V5-SBP-tagged GFP-MBP fusion served as a negative control. The inputs (0.5%) and bound fractions (3% V5 proteins and 35% CNOT1 and CNOT3) were analyzed by Western blotting. (*G*) A GST pull-down assay showing the interaction of the GST-Nanos1–3 NIMs with the recombinant NOT module, the CNOT1 SHD, and CNOT2–CNOT3 heterodimers. GST served as a negative control.

The CCR4–NOT deadenylase complex plays a central role in post-transcriptional mRNA regulation by catalyzing the removal of mRNA poly(A) tails, repressing translation, and promoting mRNA degradation (Collart and Panasenko 2012; Wahle and Winkler 2013). The CCR4–NOT complex consists of several independent modules that dock on the CNOT1 scaffold subunit (Collart and Panasenko 2012; Wahle and Winkler 2013). CNOT1 features a modular domain organization consisting of an N-terminal (CNOT1-N), a middle (CNOT1-M), and a C-terminal (CNOT1-C) region (Supplemental Fig. 1A). The CNOT1-M region interacts with the catalytic module, which comprises two deadenylases; namely, CAF1 (or its paralog, POP2) and CCR4a (or its paralog, CCR4b) (Wahle and Winkler 2013). The CNOT1-C region contains the NOT1 superfamily homology domain (SHD) (Supplemental Fig. 1A), which interacts with CNOT2–CNOT3 heterodimers to form the NOT module (Bhaskar et al. 2013; Boland et al. 2013). CNOT1 and CNOT3, in turn, serve as binding platforms for translational regulators, including Nanos and Bicaudal-C, which recruit the CCR4–NOT complex to their targets (Chicoine et al. 2007; Barckmann and Simonelig 2013).

Specifically, murine Nanos2 has been shown to directly bind to the CNOT1 subunit of the CCR4–NOT complex (Suzuki et al. 2012). The binding regions were mapped to a conserved 10-amino-acid stretch at the N terminus of Nanos2 and the C terminus of CNOT1 (Suzuki et al. 2012). Accordingly, the equivalent region in the N terminus of *Xenopus laevis* Nanos1 is necessary for the translational repression of bound mRNAs in oocytes (Lai et al. 2011).

Despite the extensive body of information regarding the biological function of Nanos proteins in diverse organisms, a detailed molecular understanding of the mechanism underlying their regulatory function remains scarce. In this study, we aimed to bridge this gap by investigating the structural basis for the recruitment of the CCR4–NOT complex by Nanos. First, we found that a short linear motif (SLiM) comprising the previously identified conserved residues (Fig. 1A,B; Supplemental Fig. 1B; Lai et al. 2011; Suzuki et al. 2012) is necessary and sufficient to mediate direct binding to the CNOT1 SHD. We termed this motif the NOT1-interacting motif (NIM). We then determined the structure of the Nanos1 NIM peptide bound to the CNOT1 SHD and identified the critical interface residues. Substitutions of these residues in Nanos1–3 prevent binding to CNOT1 and abrogate the ability of Nanos proteins to repress translation in the absence of deadenylation. These results identify the CCR4–NOT complex as a main effector complex for Nanos function. Finally, we showed that the 17-amino-acid-long NIM peptides are sufficient for the recruitment of the CCR4–NOT complex to bound mRNAs and can trigger translational repression in the absence of mRNA degradation. Our observations have important biological implications because the CCR4–NOT complex is recruited by numerous RNA-associated proteins, including GW182 (involved in the microRNA [miRNA] pathway), Bicaudal-C, Smaug, CUP, Pumilio, and tristetraprolin (TTP) (Chicoine et al. 2007; Barckmann and Simonelig 2013; Fabian et al. 2013), which suggests the existence of a common and widespread mRNA-repressive mechanism in eukaryotic cells.

## Results

### The conserved motifs in Nanos1–3 interact directly with the CNOT1 SHD

To investigate whether the three human Nanos paralogs interact with the CCR4–NOT complex, we expressed the proteins with a V5-SBP (streptavidin-binding peptide) tag in HEK293T cells and tested their interaction with the endogenous CCR4–NOT complex using SBP pull-down assays. All three Nanos proteins pulled down endogenous CNOT1 and CNOT3 (Fig. 1C–E, lane 6). Moreover, the deletion of the conserved motif at the N termini of all three Nanos (Nanos1 Δ40–56, Nanos2 Δ6–22, and Nanos3 Δ4–20) abolished the interactions (Fig. 1C–E, lanes 8), which is in agreement with the results reported for murine Nanos2 (Suzuki et al. 2012). Remarkably, the conserved motifs of Nanos2 and Nanos3 (fused to V5-SBP-MBP for detection) were sufficient for the interaction with endogenous CNOT1 and CNOT3 (Fig. 1D,E, lanes 7). In contrast, the isolated conserved motif of Nanos1 failed to interact with CNOT1 and CNOT3 (Fig. 1C, lane 7). Further studies using longer Nanos1 fragments indicated that the extension of the conserved motif to the very N terminus of the protein (Nanos1 fragment 1–58) was sufficient to observe binding to endogenous CNOT1 and CNOT3 (Fig. 1F, lane 7).

Murine Nanos2 interacts directly with a C-terminal region of CNOT1 (corresponding to residues 1586–2376) (Supplemental Fig. 1A; Suzuki et al. 2012). Our previous structural studies indicated that this region contains a NOT1 SHD (residues 1833–2361) that assembles with CNOT2 and CNOT3 to form the NOT module (Boland et al. 2013). To determine whether the CNOT1 SHD is sufficient for Nanos binding, we performed pull-down assays in vitro with recombinant proteins expressed in *Escherichia coli*. The conserved motifs of Nanos1–3 fused to glutathione S-transferase (GST) pulled down the isolated CNOT1 SHD and a preassembled NOT module containing the CNOT1 SHD together with the CNOT2 and CNOT3 C-terminal domains (Fig. 1G), indicating that the motifs interact directly with the CNOT1 SHD in both isolation and the context of the NOT module. The motifs were therefore termed NIMs. No direct interaction was observed with the isolated CNOT2–CNOT3 subcomplex, suggesting that these two proteins are not required for the binding of the NIMs to the NOT module (Fig. 1G).

Importantly, the Nanos1 NIM was sufficient for the interaction with CNOT1 under these conditions. However, the pull-down efficiency was consistently reduced compared with the binding of Nanos2 and Nanos3, which provides an explanation for the lack of interaction under the more stringent conditions of the pull-down assay in cell lysates (Fig. 1C).

### The NIMs are required for Nanos-mediated mRNA degradation

**Figure 2.**
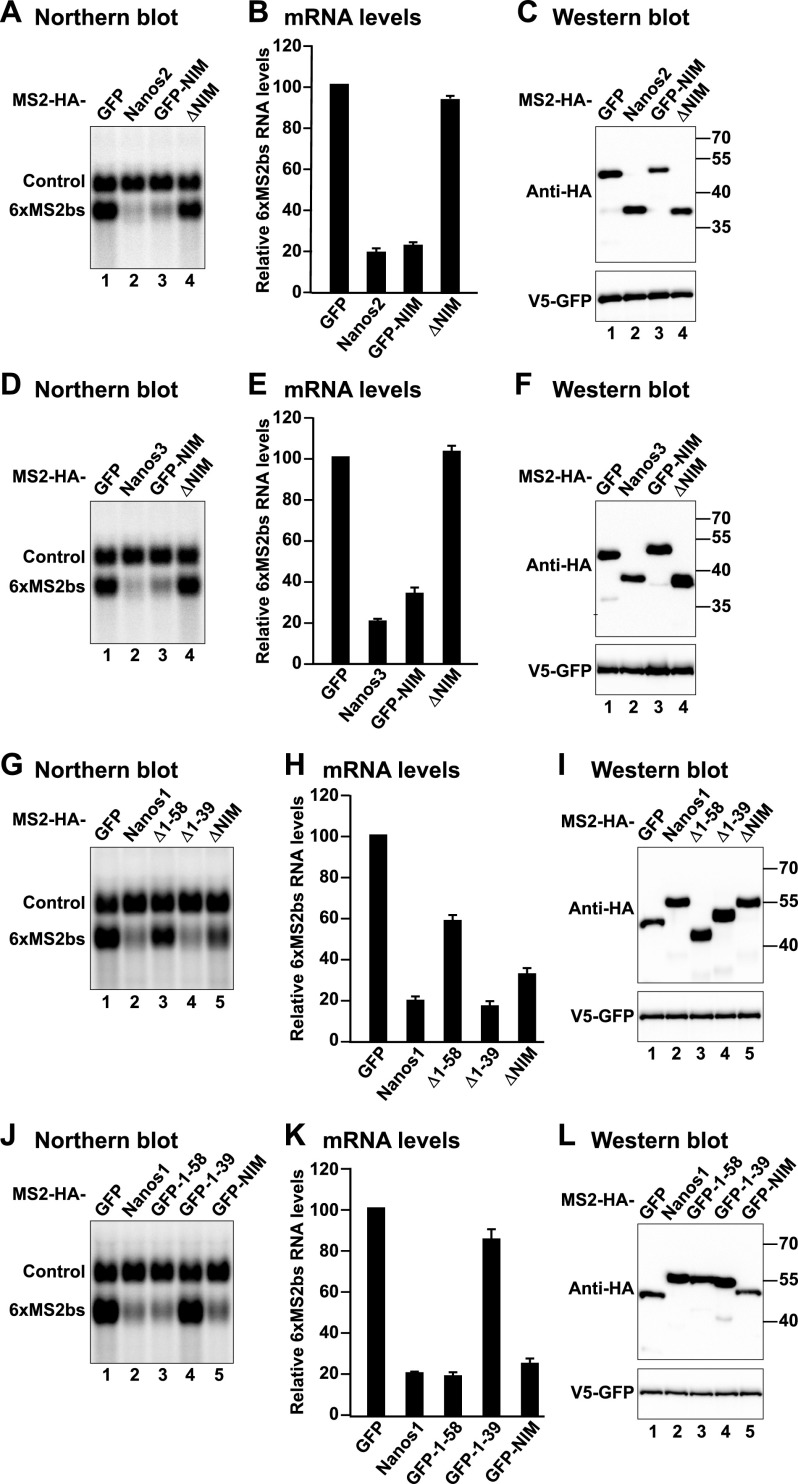
Nanos1–3 NIMs cause degradation of bound mRNAs. (*A–L*) Tethering assays using the β-globin-6xMS2bs reporter and the indicated MS2-HA-tagged proteins. A plasmid expressing an mRNA lacking MS2-binding sites (control) served as a transfection control. The β-globin-6xMS2bs mRNA levels were normalized to those of the control mRNA and set to 100 in the presence of MS2-HA-GFP. The mean values ± standard deviations from three independent experiments are shown in *B*, *E*, *H*, and *K*. (*A*,*D*,*G*,*J*) Northern blots of representative RNA samples. (*C*,*F*,*I*,*L*) Western blot analysis showing the equivalent expression of the MS2-HA-tagged proteins used in the corresponding tethering assays.

To investigate the relevance of the NIM for Nanos function, we tethered a MS2-tagged Nanos protein to a β-globin reporter containing six MS2-binding sites in the 3′ untranslated region (UTR) (β-globin-6xMS2bs) (Lykke-Andersen et al. 2000). This tethering assay allowed us to study the intrinsic activity of Nanos independently of Pumilio. The tethered Nanos proteins reduced the β-globin-6xMS2bs mRNA levels relative to the MS2-HA-GFP fusion protein, which was used as a negative control (Fig. 2A–L). The reduction in mRNA levels was explained by a shortening of the mRNA half-life (Supplemental Fig. 1C), indicating that Nanos proteins induce mRNA degradation. The levels of the control β-globin mRNA lacking MS2-binding sites were not affected (Fig. 2A,D,G,J, control).

The deletion of the NIMs in Nanos2 and Nanos3 abrogated the ability of the proteins to degrade the mRNA reporter (Fig. 2A,D [cf. lanes 4 and 2], B,E), indicating that the NIMs are strictly required for mRNA degradation mediated by Nanos2 and Nanos3. In contrast, the deletion of the Nanos1 NIM alone or together with the N-terminal extension (residues 1–58) reduced but did not abolish the activity of the protein in tethering assays (Fig. 2G), suggesting that additional sequences in Nanos1 contribute (directly or indirectly) to the recruitment of effector complexes.

Strikingly, the isolated Nanos1–3 NIMs fused to MS2-HA-GFP were sufficient to trigger mRNA target degradation (Fig. 2A,D [lane 3], J [lane 5], B,E,K). In contrast, the residues of Nanos1 upstream of the NIM (residues 1–39) did not trigger target degradation (Fig. 2J [lane 4], K). Furthermore, the deletion of residues 1–39 in Nanos1 was ineffectual in this assay (Fig. 2G [cf. lanes 4 and 2], H). Thus, the Nanos1 NIM is sufficient for the recruitment of the CCR4–NOT complex to the mRNA target in a cellular context, whereas the upstream N-terminal 1–39 residues may play an auxiliary role in facilitating the interaction with the CCR4–NOT complex but are not sufficient for binding on their own. The Nanos proteins and the corresponding fragments were expressed at comparable levels (Fig. 2C,F,I,L).

**Figure 3.**
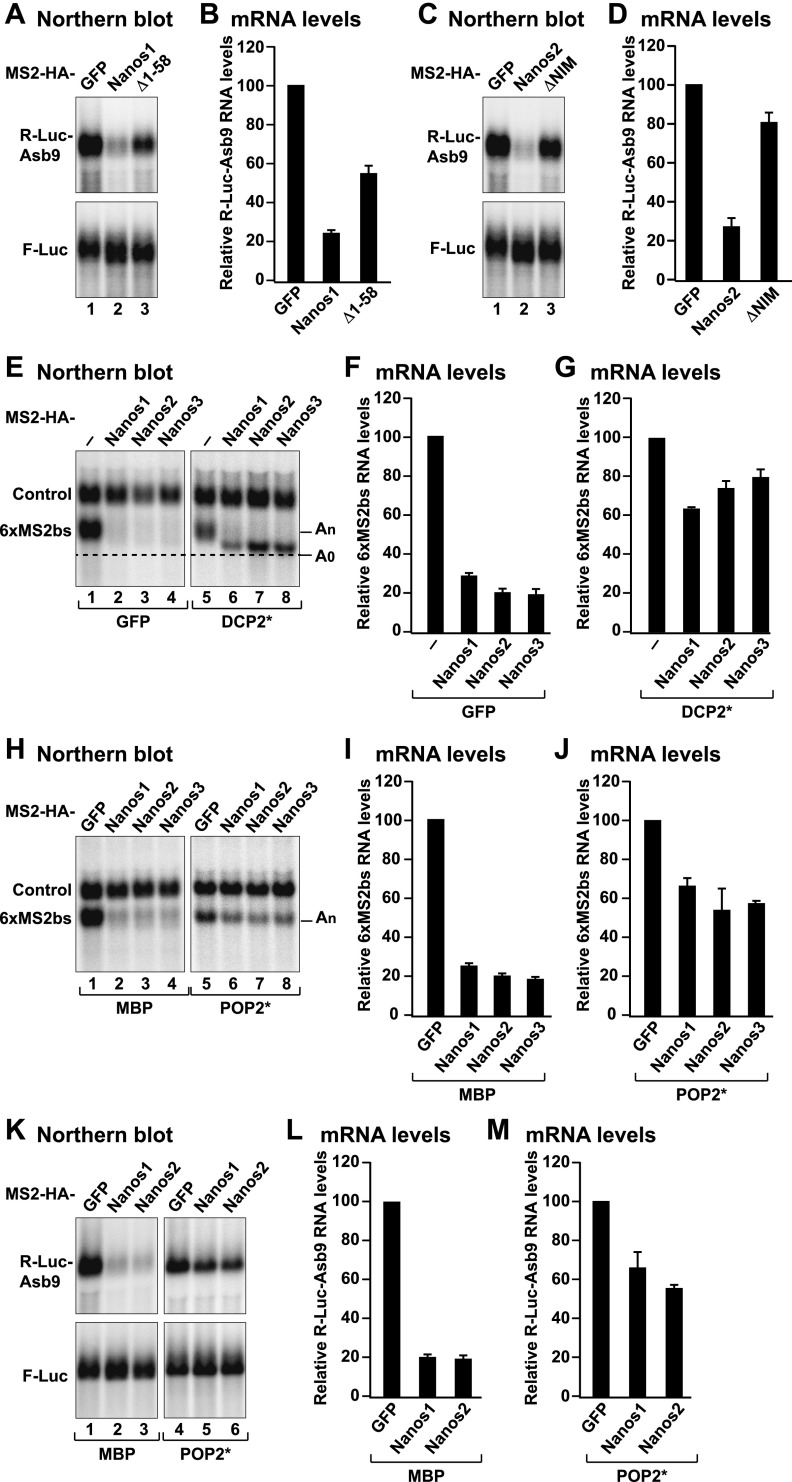
Nanos1–3 promote deadenylation-dependent decapping. (*A–D*) mRNA degradation assay using the R-Luc-Asb9 reporter (lacking MS2-binding sites) in cells coexpressing the indicated MS2-HA-tagged proteins. A plasmid expressing F-Luc mRNA served as a transfection control. The R-Luc-Asb9 mRNA levels were normalized to the control mRNA and set to 100 in the presence of MS2-HA-GFP. The mean values ± standard deviations from three independent experiments are shown in *B* and *D*. (*A*,*C*) Northern blots of representative RNA samples. (*E–J*) Tethering assays using the β-globin-6xMS2bs reporter were performed as described in Figure 2 with the exception that plasmids expressing the DCP2* or POP2* catalytically inactive mutants were included in the transfection mixtures as indicated. (*E*,*H*) Northern blots of representative RNA samples. (*F*,*G*,*I*,*J*) Normalized levels of the β-globin-6xMS2bs mRNA. The expression of the DCP2* and POP2* proteins is shown in Supplemental Figure 2, G and H. (*K–M*) The effect of Nanos1 and Nanos2 on the expression of the R-Luc-Asb9 mRNA reporter was tested as described in *A–D* with the exception that a plasmid expressing the POP2* catalytically inactive mutant was included in the transfection mixtures as indicated.

The results of the tethering assays were validated using a reporter containing the *Renilla* luciferase (R-Luc) ORF fused to the 3′ UTR of the Asb9 (ankyrin repeat and SOCS box containing 9) mRNA, which was previously shown to be a target of murine Nanos2 in male embryonic gonads (Suzuki et al. 2010). Both Nanos1 and Nanos2 reduced the expression of the R-Luc-Asb9 reporter in HEK293T cells (Fig. 3A–D; Supplemental Fig. 2A,B), indicating that the proteins are capable of binding to the Asb9 3′ UTR and that HEK293T cells provide the cofactors required for their repressive activity. The reduction in R-Luc activity was accompanied by a corresponding decrease in the R-Luc-Asb9 mRNA levels (Fig. 3A,C [lanes 2], B,D). The repression of the R-Luc-Asb9 reporter was also observed when the MS2 tag was replaced by GFP and was dependent on the presence of the zinc finger domains (Supplemental Fig. 2C–F), further supporting the conclusion that Nanos1 and Nanos2 bind directly to the Asb9 3′ UTR. Similar to the results obtained in the tethering assays, the ability of Nanos2 to trigger R-Luc-Asb9 mRNA degradation was abolished by the deletion of the NIM (Fig. 3C,D), whereas Nanos1 exhibited residual activity after the deletion of all of the N-terminal residues (Δ1–58) (Fig. 3A,B).

In contrast, Nanos3 had no effect on the R-Luc-Asb9 reporter (Supplemental Fig. 2A,B). Because Nanos3 down-regulates the expression of a reporter when it is directly tethered, it is likely that its inability to repress the R-Luc-Asb9 reporter is caused by a lack of binding affinity for the Asb9 3′ UTR.

### Nanos proteins trigger deadenylation-dependent decapping

Although the mechanism of Nanos-mediated mRNA-degradation has not been investigated in detail, a reasonable assumption would be that Nanos proteins trigger deadenylation by interacting with the CCR4–NOT complex. Deadenylation is typically coupled to decapping and 5′-to-3′ exonucleolytic degradation by XRN1 in many cellular contexts (Wahle and Winkler 2013). We therefore investigated whether Nanos proteins trigger deadenylation-dependent decapping.

If deadenylation precedes decapping and 5′-to-3′ mRNA degradation, then deadenylated mRNA decay intermediates are expected to accumulate in cells in which decapping is inhibited. Consistent with this expectation, the degradation of the β-globin-6xMS2bs reporter by Nanos1–3 was inhibited in cells overexpressing a catalytically inactive DCP2 mutant (E148Q mutant termed DCP2*) (Fig. 3E–G; Supplemental Fig. 2G). The reporter accumulated in a fast-migrating form, corresponding to the deadenylated decay intermediate (A_0_). This assumption was confirmed using RNase H cleavage assays (Supplemental Fig. 2I). Thus, Nanos1–3 elicit mRNA decay by triggering deadenylation and then decapping. Accordingly, the ability of Nanos proteins to elicit the degradation of the β-globin-6xMS2bs and R-Luc-Asb9 mRNAs was impaired in cells overexpressing a catalytically inactive POP2 mutant (D40A, E42A mutant termed POP2*) (Fig. 3H–M; Supplemental Fig. 2H).

### The NIMs are sufficient for Nanos-mediated translational repression in the absence of mRNA deadenylation

*D. melanogaster* Nanos promotes deadenylation and represses translation in the absence of mRNA degradation during oogenesis and early embryogenesis (Wharton and Struhl 1991; Wreden et al. 1997; Kadyrova et al. 2007). Furthermore, Nanos proteins can repress translation in the absence of deadenylation (Chagnovich and Lehmann 2001; Lai et al. 2011). To investigate whether human Nanos proteins can repress translation in the absence of mRNA deadenylation and decay, we generated a MS2 reporter containing the R-Luc ORF fused to the 3′ end of the noncoding RNA MALAT1. The 3′ end of the MALAT1 RNA is generated through endonucleolytic cleavage by RNase P and thus is not polyadenylated (Wilusz et al. 2012). Nevertheless, this 3′ UTR promotes nuclear export and supports efficient translation (Wilusz et al. 2012).

**Figure 4.**
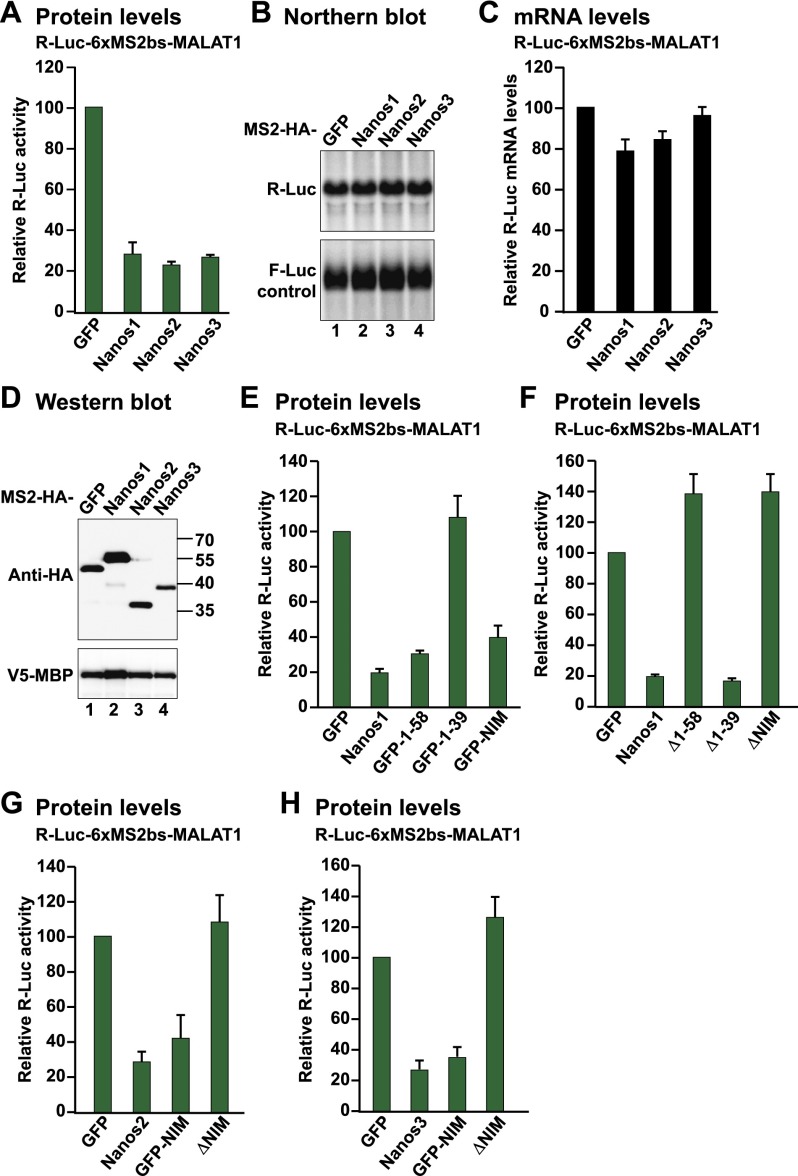
Nanos1–3 NIMs repress translation in the absence of mRNA degradation. (*A–H*) Tethering assay using the R-Luc-6xMS2bs-MALAT1 reporter and the indicated MS2-HA-tagged proteins. A plasmid expressing F-Luc served as a transfection control. The R-Luc activities and mRNA levels were normalized to those of the F-Luc transfection control and set to 100 in the presence of MS2-HA-GFP. (*A*,*E*–*H*) Normalized R-Luc activities obtained in three independent experiments. (*B*) Northern blot of representative RNA samples. The Northern blots corresponding to the samples shown in *G* and *H* are shown in Supplemental Figure 2K. (*C*) Normalized R-Luc mRNA levels. The mean values ± standard deviations from three independent experiments are shown. (*D*) Western blot analysis showing the expression of the MS2-HA-tagged proteins.

The three Nanos proteins repressed the expression of the R-Luc-6xMS2bs-MALAT1 reporter without causing corresponding changes in mRNA levels (Fig. 4A–D). This repression was fully dependent on the NIMs for all three Nanos proteins (Fig. 4E–H; Supplemental Fig. 2J,K). Indeed, no additional translational-repressive activity was observed for the Nanos1 protein lacking the NIM (Fig. 4F). Remarkably, the NIMs fused to MS2-HA-GFP were sufficient to cause translational repression in the absence of mRNA degradation (Fig. 4E–H; Supplemental Fig. 2J,K), whereas Nanos1 residues 1–39 had no repressive activity (Fig. 4E). We thus conclude that the NIMs are the major determinants for the translational repression mediated by Nanos in the absence of mRNA deadenylation and decay.

It is important to note, however, that Nanos proteins associate with additional mRNA regulators such as Pumilio and Brain Tumor depending on the cellular context and the mRNA target (Sonoda and Wharton 1999, 2001; Asaoka-Taguchi et al. 1999; Jaruzelska et al. 2003) and thus can potentially repress translation indirectly in a NIM-independent manner.

### Structure of the Nanos1 NIM peptide bound to CNOT1

**Figure 5.**
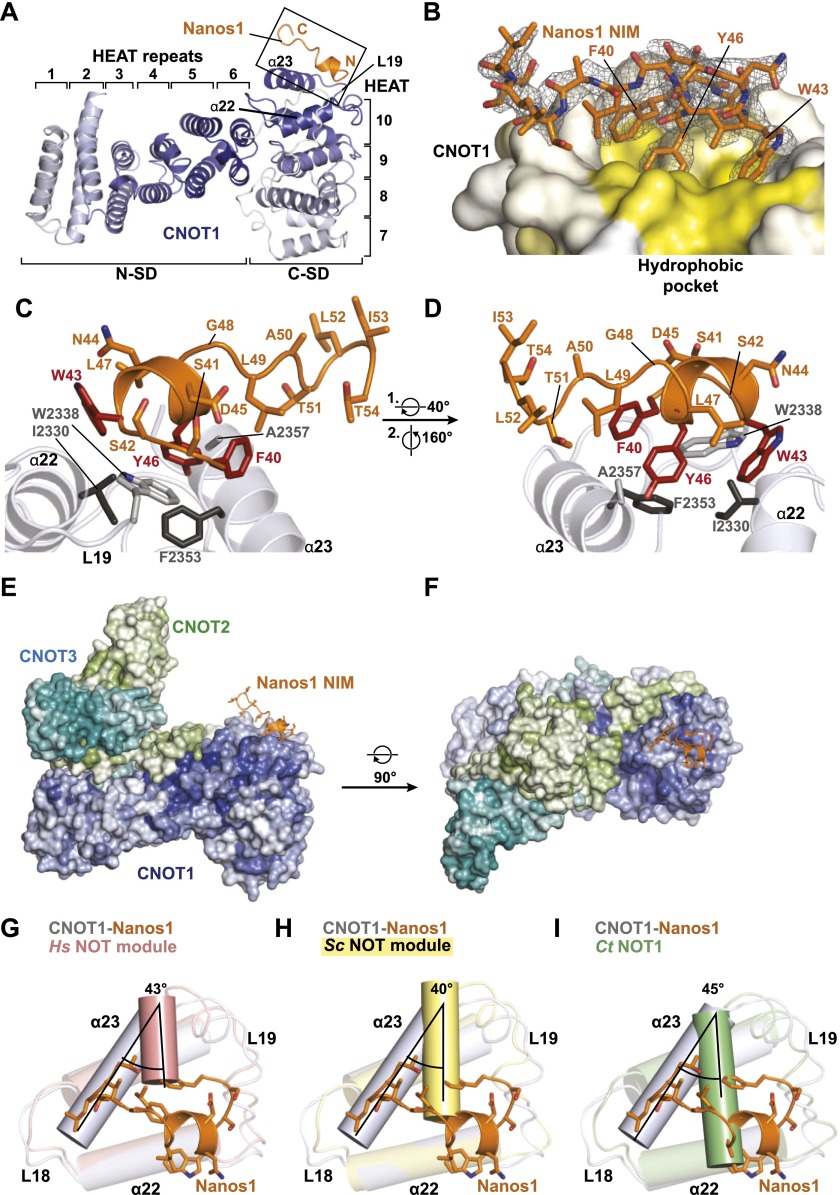
Structure of CNOT1–Nanos1 NIM complex. (*A*) Cartoon representation of the Nanos1 peptide (orange) bound to the CNOT1 SHD. The HEAT-like repeats that form the CNOT1 SHD are numbered. Both N-SD and C-SD (N-terminal and C-terminal subdomains, respectively) are colored in a gradient from gray to blue from their N termini to the C termini, respectively. (*B*) Surface representation of the Nanos-binding pocket of CNOT1 colored in a gradient from white to yellow with increasing hydrophobicity. The electron difference density for the Nanos1 NIM peptide (chain B) is shown as a black mesh (difference density [F_0_ − F_C_] contoured at 2.0 σ using the refined CNOT1 model before the NIM peptide was built), and the corresponding structural model is displayed as orange sticks. (*C*,*D*) Close-up views of the binding interface. The residues of CNOT1 and the Nanos1 peptide are shown as gray and orange sticks, respectively. The residues of CNOT1 and Nanos1 mutated in this study are highlighted in dark gray and red, respectively. (*E*) Structural model showing the Nanos1 NIM peptide bound to the human NOT module. The model was created by the superposition of the human NOT module (Boland et al. 2013) onto the CNOT1–Nanos1 structure. CNOT1 from the CNOT1–Nanos1 structure and CNOT2–CNOT3 from the NOT module are shown in surface representation. Nanos1 is represented as a cartoon (orange). The conservation of surface residues in the NOT module is indicated by color gradients from light (no conservation) to dark (100% conservation) for CNOT1 (blue), CNOT2 (green), and CNOT3 (cyan). The conservation scores were calculated based on well-balanced multiple alignments covering all eukaryotic strata. (*G–I*) Conformational change of the CNOT1 α23 helix in the complex with the Nanos1 NIM peptide. The CNOT1 SHD structures from the human and yeast NOT modules and the *C. thermophilum* (*Ct*) NOT1 SHD are shown in salmon (*G*), yellow (*H*), and green (*I*), respectively. The CNOT1 SHD bound to the Nanos1 NIM peptide is shown in gray, and the Nanos1 NIM peptide is shown in orange. The black lines illustrate the orientation of the α23 helix in the two structures. The angle between the two conformations is indicated. PDB codes are as follows: 4C0D (human NOT module), 4BY6 (*Saccharomyces cerevisiae* NOT module), and 4C0E (*C. thermophilum* NOT1).

Given the relevance of the NIMs for Nanos function, we sought to determine the molecular details of the interaction with the CNOT1 SHD. To this end, we crystallized the CNOT1 SHD (residues 1833–2361) (Boland et al. 2013) in complex with the NIM peptide of Nanos1 (residues 40–56). No crystals could be obtained with the Nanos2 or Nanos3 NIM peptides. The structure was solved by molecular replacement using the structure of the human CNOT1 SHD (Protein Data Bank [PDB] code 4C0D) (Boland et al. 2013) as the search model and refined at a resolution of 2.8 Å (Table 1; Fig. 5; Supplemental Fig. 3).

**Table 1.**
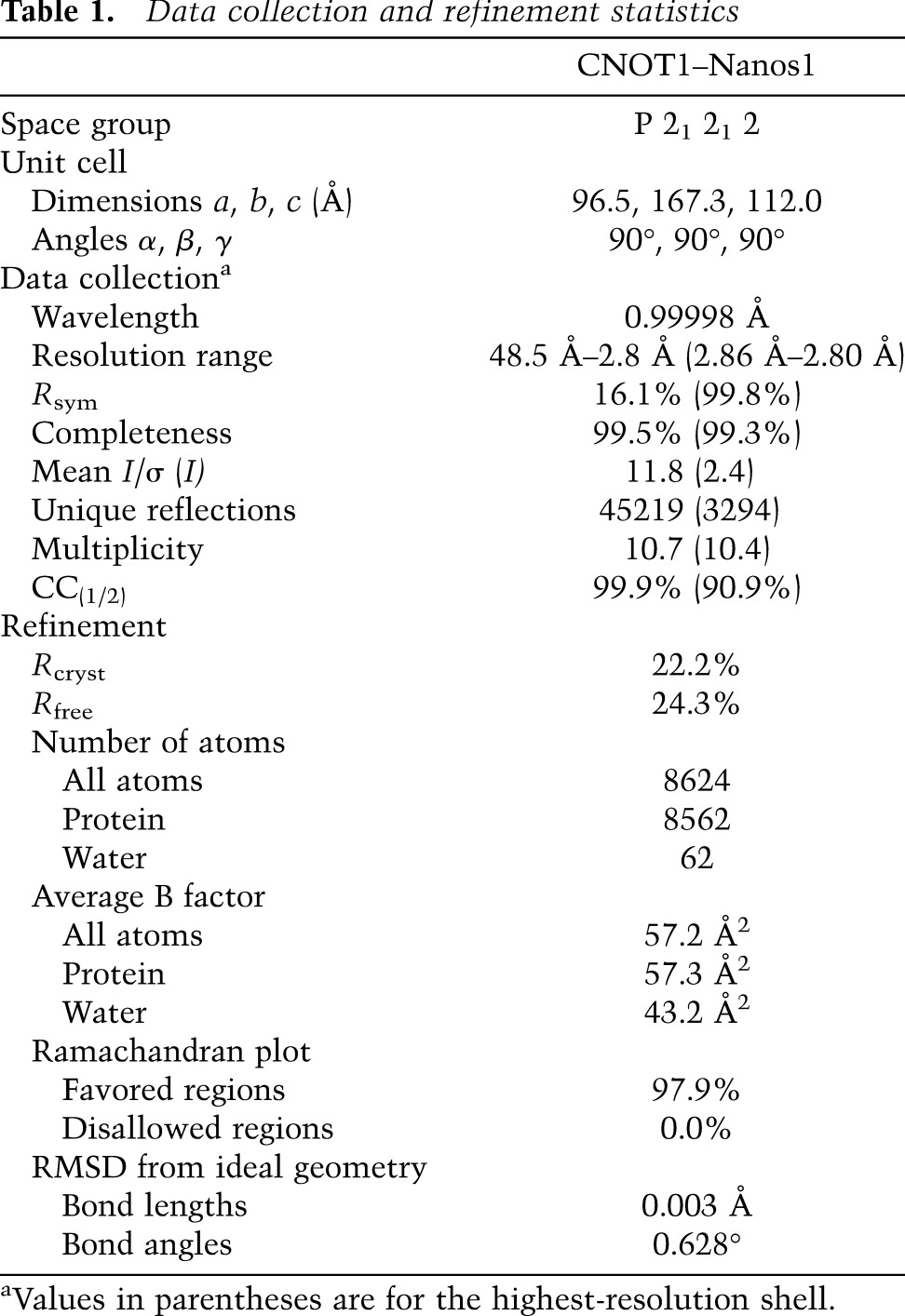
Data collection and refinement statistics

The CNOT1–Nanos1 crystals contain two molecules of CNOT1 sandwiching two Nanos1 peptides between them (Supplemental Fig. 3A). The peptides bridge the CNOT1 molecules and mediate important crystal-packing contacts via residues that are divergent between the three Nanos paralogs (Supplemental Fig. 3A,B), which explains why crystals with the Nanos2 and Nanos3 NIMs could not be obtained under similar conditions. Most residues of CNOT1 (1842–2361), with the exception of two surface loops (residues 1917–1924 and 2083–2084 in chain A and residues 1922–1925 in chain C), are visible in the electron density. The electron densities for both Nanos1 peptides in the asymmetric unit are well defined and can be modeled with residues Phe40–Thr54 (chain B) and Phe40–Ile53 (chain D), respectively (Fig. 5B). Because the structures of the two monomers of CNOT1 and the Nanos1 molecules are virtually identical (the RMSD is 0.15 Å over 420 Cα for CNOT1 and 0.11 Å over 13 Cα for Nanos1) (Supplemental Fig. 3C,D), we focus our description of the CNOT1–Nanos1 structure on the complex with the better defined NIM (PDB chains A and B).

### The CNOT1–Nanos1 interface

The fold of the CNOT1 SHD has been described previously and consists of 23 α helices arranged in a series of HEAT-like repeats that are organized into two perpendicular stacks to form the N-terminal and the C-terminal subdomains (N-SD and C-SD, respectively) (Fig. 5A, Boland et al. 2013). Due to the packing of the molecules in the crystal, CNOT1 is in contact with two Nanos1 peptides. However, one of the contacts (chain D) (Supplemental Fig. 3A) is less extensive, is mediated by nonconserved residues, and overlaps entirely with the binding site of CNOT3 on the CNOT1 surface (Supplemental Fig. 3E,F). Therefore, this contact can be excluded as the real NIM-binding site because the NIM peptides interact with CNOT1 in the context of the NOT module.

The other Nanos1 peptide (chain B) contacts the helices of the final HEAT-like repeat of CNOT1 (chain A, α22 and α23) (Fig. 5A; Supplemental Fig. 3A). The superposition of this complex with the CNOT1–CNOT2–CNOT3 structure (Boland et al. 2013) indicates that this surface is also available for Nanos proteins in the context of the NOT module (Fig. 5E,F).

Although the overall arrangement of CNOT1 in complex with Nanos1 does not change compared with the arrangement of CNOT1 in the NOT module (RMSD of 0.91 Å over 420 Cα) (Supplemental Fig. 3G), an important difference is evident in the last α helix (α23) of the protein. In the structures of the human and yeast NOT modules and the isolated *Chaetomium thermophilum* NOT1 SHD, helix α23 partially occludes the Nanos-binding site (Fig. 5G––I; Supplemental Fig. 3G,H, cf. positions of the vertical salmon, yellow, and green helices; Bhaskar et al. 2013; Boland et al. 2013). Because this helix arrangement is conserved in three independent NOT1 structures from three different organisms, it is unlikely to be the result of crystal contacts. In contrast, helix α23 displays two additional turns in the structure of the CNOT1–Nanos1 complex (Fig. 5G–I gray helix) and moves by 43° relative to its position in the human NOT module (Fig. 5G, salmon helix), which results in the opening up of the Nanos-binding site (Fig. 5G–I; Supplemental Fig. 3G,H). This observation suggests that NIM binding is accompanied by a mutually induced fit of CNOT1 and Nanos and is not the result of a simple docking into a completely preformed pocket.

The CNOT1 residues lining the Nanos-binding site are well conserved and are mainly hydrophobic (Fig. 5B–D; Supplemental Fig. 4). The first eight residues of the Nanos1 peptide form a turn and a short α helix that lies almost perpendicular on top of the CNOT1 helix α23 (Fig. 5C,D), thereby covering the hydrophobic pocket (Fig. 5B). The hydrophobicity of the pocket is matched by the conserved hydrophobic NIM residues that mediate the interaction.

More specifically, three highly conserved aromatic residues (namely, F40, W43, and Y46) insert deep inside the CNOT1 pocket, where Y46 forms a hydrogen bond with the backbone oxygen of CNOT1 F2353 (Fig. 5C,D). The orientation of the turn is fixed by the invariant residue D45, which provides two intramolecular hydrogen bonds (one to the backbone amide nitrogen and one to the hydroxyl group of S41) (Fig. 5D). These interactions tether F40 to the side of the short helix and allow it to pack into the fairly small CNOT1 pocket alongside W43 and Y46 (Fig. 5D).

In essence, the conformation adopted by the peptide backbone allows the simultaneous arrangement of the aromatic residues along the hydrophobic cavity and maximizes the interactions with the nonconserved residues through main chain interactions, such as those observed between S41 and S42 of the peptide and W2338 of CNOT1. The other invariant NIM residue, L49, is located at the end of the short helix. This residue makes hydrophobic contacts with A2357 of CNOT1 and packs against Nanos1 F40 and Y46, which most likely results in the stabilization of the overall conformation of the peptide (Fig. 5D).

The C-terminal portion of the peptide (residues 50–54) is elongated and forms a turn that is stabilized by intramolecular backbone hydrogen bonds (Fig. 5B–D). Consistent with its lower conservation (Fig. 1B), this portion of the peptide makes only minor contacts to the CNOT1 molecule in the crystal. Furthermore, the higher B factors indicate the greater flexibility of this part of the peptide.

Sequence comparisons indicate that the NIM is present in Nanos proteins from a diverse set of metazoan phyla ranging from sponges, coelenterates, and mollusks to chordates, hemichordates, and echinodermata (Supplemental Fig. 5A; Lai et al. 2011; Suzuki et al. 2012). However, the NIM sequence could not be detected in any insects, flat worms, annelids, or rotifera Nanos proteins. Interestingly, *D. melanogaster* Nanos does not contain a NIM even though it recruits the CCR4–NOT complex (Joly et al. 2013; D Bhandari and E Izaurralde, unpubl.). The alignment of NIM sequences from all NIM-containing Nanos proteins revealed that the residues at positions 1 (F), 4 (W), 6 (D), 7 (Y), and 10 (L) are almost invariant (Supplemental Fig. 5B), consistent with their important role in mediating CNOT1 binding. Because the hydrophobic binding pocket in CNOT1 is also highly conserved (Supplemental Fig. 4), this observation suggests that the mode of CCR4–NOT recruitment is conserved for all Nanos proteins containing a NIM.

### Validation of the interface

**Figure 6.**
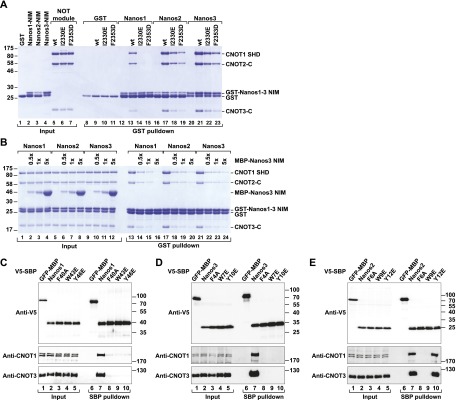
Validation of the interaction interface. (*A*) A GST pull-down assay showing the interaction of the GST-Nanos1–3 NIMs with the recombinant NOT module containing the wild-type CNOT1 SHD or the indicated CNOT1 mutants. GST served as a negative control. (*B*) A GST pull-down assay showing that the MBP-tagged Nanos3 NIM peptide competes with the GST-tagged Nanos1–3 NIMs for binding to the NOT module. The MBP-Nanos 3 NIM competitor was present in 0.5-fold, onefold, and fivefold molar excess relative to the GST-NIMs. The corresponding experiment showing that the Nanos3 NIM Y10E mutant does not compete for binding is shown in Supplemental Figure 6A. (*C–E*) Interaction of V5-SBP-tagged Nanos1–3 (wild type or mutants) with endogenous CNOT1 and CNOT3 in HEK293T cells.

To test whether CNOT1 and the Nanos1 NIM interact in solution in a manner similar to that observed in the crystal structure, we substituted CNOT1 conserved residues I2330 and F2353, which are located at the center of the interface, with glutamate and aspartate. These substitutions did not affect the interaction of CNOT1 with CNOT2 and CNOT3 (Fig. 6A, lanes 5–7), indicating that the substitutions do not alter the SHD fold. The individual I2330E and F2353D substitutions were sufficient to abolish the binding of the Nanos1 NIM and reduced the interaction with the Nanos2 and Nanos3 NIMs (Fig. 6A, lanes 13–23), indicating that all NIMs bind to the same surface on CNOT1. However, the residual binding displayed by the Nanos2 and Nanos3 NIMs suggests that they may form more extensive interactions with the CNOT1 surface by establishing additional contacts with paralog-specific residues.

In agreement with the hypothesis that all three NIMs dock on the same hydrophobic pocket of CNOT1, we observed that the Nanos3 NIM peptide competes with the binding of Nanos1–3 NIMs to the NOT module in vitro (Fig. 6B, lanes 13–24). The ability to compete was suppressed by a Y10E substitution (Supplemental Fig. 6A), which abolished the binding of Nanos3 to CNOT1 (Fig. 6D; see below).

To further validate the CNOT1–Nanos interface, we substituted the three conserved aromatic residues F40, W43, and Y46 in the context of full-length Nanos1 and tested the interaction with the CCR4–NOT complex using SBP pull-down assays in HEK293T cells. The individual F40A, W43E, and Y46E substitutions were sufficient to abolish the interaction of Nanos1 with endogenous CNOT1 and CNOT3 (Fig. 6C, lanes 7–10). Similarly, the corresponding substitutions in Nanos3 (F4A, W7E, and Y10E) abrogated its interaction with endogenous CNOT1 and CNOT3 (Fig. 6D, lanes 7–10). In Nanos2, the corresponding F6A and W9E substitutions also abolished the interaction, but the Y12E substitution was ineffectual (Fig. 6E). It is possible that the divergent residues of the Nanos2 NIM make additional contacts to CNOT1, thereby decreasing the influence of the Y12 residue on CNOT1 binding.

In summary, our results indicate that the NIMs of Nanos1–3 compete for binding to the same hydrophobic pocket in the CNOT1 SHD. In particular, the conserved F and W residues in the NIMs play an essential role in the interaction and most likely contact CNOT1 using a similar binding mode, whereas the contributions of the nonconserved residues likely differ between the motifs.

### The interaction with CNOT1 is essential for translational repression by Nanos

**Figure 7.**
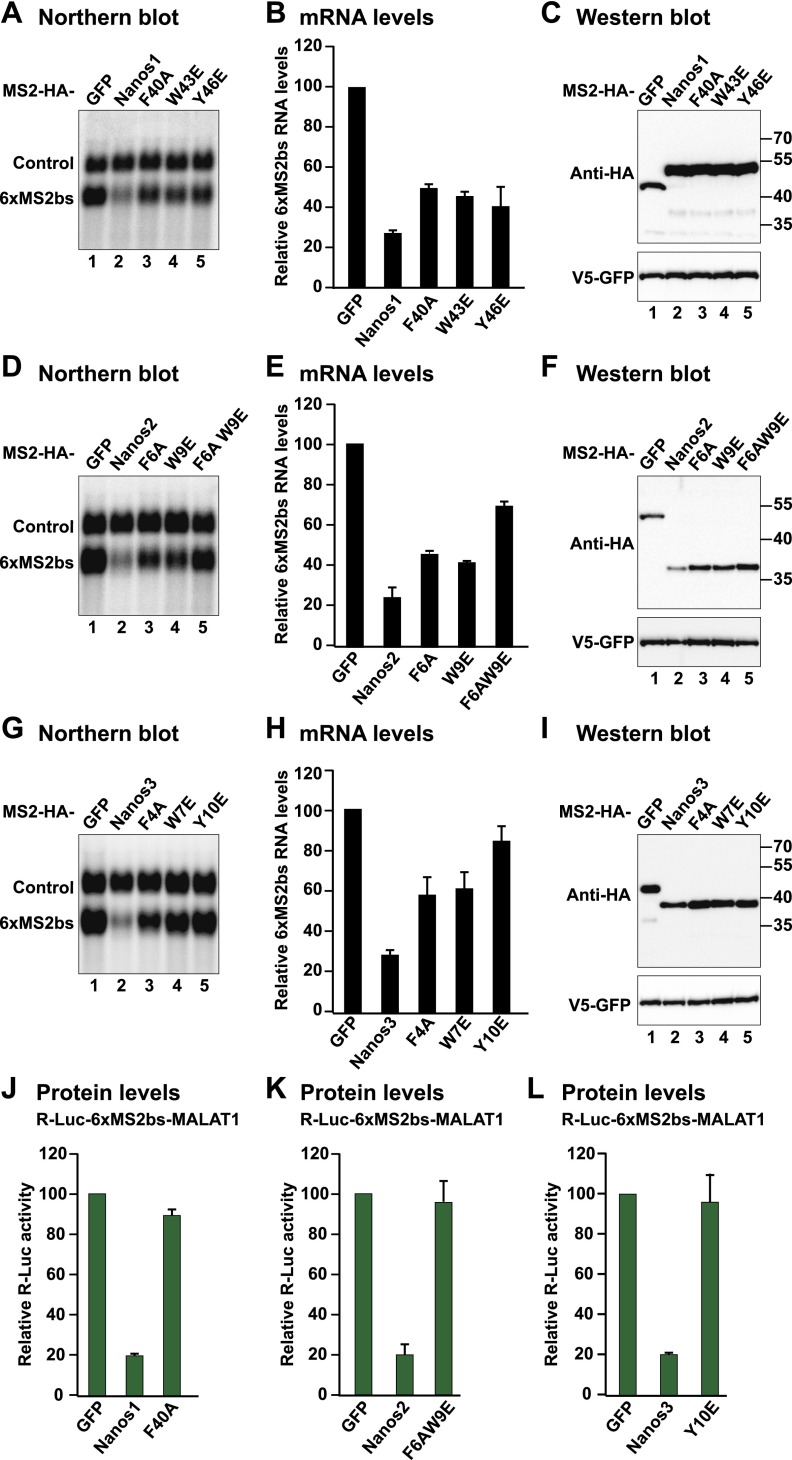
Mutations in the NIMs abrogate translational repression mediated by Nanos1–3. (*A–I*) Tethering assays using the β-globin-6xMS2bs reporter and the indicated proteins were performed as described in Figure 2. (*A*,*D*,*G*) Northern blots of representative RNA samples. The levels of β-globin-6xMS2bs mRNA were normalized to those of the control and set to 100 in cells expressing MS2-HA-GFP. The mean values ± standard deviations of three independent experiments are shown in *B*, *E*, and *H*. (*C*,*F*,*I*) Western blot analysis showing the equivalent expression of the MS2-HA-tagged proteins used in the corresponding tethering assays. (*J–L*) Tethering assays using the R-Luc-6xMS2bs-MALAT1 reporter and the indicated proteins were performed as described in Figure 4.

To investigate the functional relevance of the interfaces described in this study for Nanos function, we tested the effect of the mutations in the conserved motifs through tethering assays. In Nanos1, the individual F40A, W43E, and Y46E substitutions reduced but did not abolish the activity of the protein in tethering assays (Fig. 7A–C), similar to the effect of the deletion of the entire N-terminal fragment (Fig. 2G) and in accordance with the possibility that additional sequences in Nanos1 recruit decay factors. In Nanos2, the individual F6A and W9E substitutions reduced the activity of the protein (Fig. 7D–F). In contrast, the Y12E substitution was ineffectual (Supplemental Fig. 6B–D), consistent with the ability of this mutant to bind CNOT1 (Fig. 6E). The Nanos2 activity was further impaired by the combination of the F6A and W9E substitutions (Fig. 7D–F). In Nanos3, all of the substitutions reduced the activity of the protein, although the Y10E substitution was more effective (Fig. 7G–I). Interestingly, alanine substitutions of the conserved phenylalanine and tyrosine residues in the NIM of *X. laevis* Nanos1 (F7A and Y13A) were previously shown to abolish the repressive activity of the protein (Lai et al. 2011), indicating that the NIMs play a conserved role in Nanos function.

We then tested the effect of the mutations on the translational repression of the MALAT1 reporter, which is strictly dependent on the NIMs, as shown in Figure 4. Strikingly, the single substitutions in the Nanos1 and Nanos3 NIMs and the double F6A/W9E substitution in Nanos2 abrogated the ability of the proteins to repress the translation of this reporter in the absence of mRNA degradation (Fig. 7J–L), further confirming the essential role of the NIMs in Nanos-mediated translational repression.

## Discussion

In this study, we elucidate the structural mechanism by which a conserved motif in vertebrate Nanos paralogs (termed NIM) recruits the CCR4–NOT complex to repress translation in the absence of mRNA degradation and promote degradation of bound mRNAs. The NIM displays many features of a SLiM (or eukaryotic linear motif [ELM]) involved in protein–protein interactions (Davey et al. 2012). Indeed, the NIM is a short 17-amino-acid motif present in the N-terminal disordered region of Nanos proteins. Unlike the surrounding residues, its sequence conservation is high, and it is enriched in aromatic and hydrophobic residues and depleted of small, polar groups.

The specific features of SLiMs have important implications on complex assembly. First, SLiMs mediate relatively low-affinity interactions. Nonetheless, the interactions can be highly specific, and high affinity can be achieved through avidity effects generated by contributions from the flanking disordered regions that extend the interaction interface (Davey et al. 2012; Tompa 2012). For example, the Nanos1 sequences located upstream of the NIM (residues 1–39) may provide additional low-affinity contacts with the CCR4–NOT complex. Alternative mechanisms to achieve high affinity include interactions with other components in large protein complexes. For instance, Nanos proteins interact with Pumilio proteins, which have also been reported to interact with the CCR4–NOT complex (Sonoda and Wharton 1999; Goldstrohm et al. 2006; Van Etten et al. 2012; Joly et al. 2013).

A second consequence of the nature of SLiMs is their evolutionary plasticity (Davey et al. 2012; Tompa 2012). Due to their shortness and lack of sequence constraints in the absence of a protein fold, even single-point mutations can render an existing motif nonfunctional or generate a new motif in another protein. We observe this plasticity in the case of Nanos; i.e., several phyla appear to have completely lost the NIM (Supplemental Fig. 5). In particular, Nanos proteins from insects and worms do not have a detectable NIM (Supplemental Fig. 5; Lai et al. 2011). Nevertheless, *D. melanogaster* Nanos has been shown to also recruit the CCR4–NOT complex using its unstructured N terminus (Kadyrova et al. 2007), indicating that the overall principle of the recruitment of the CCR4–NOT complex to mediate mRNA target repression is likely maintained even though the interaction details have changed.

The importance of the NIM and the interaction with the CCR4–NOT complex for Nanos function in vivo is further supported by the following observations. First, a murine Nanos2 variant lacking the NIM fails to rescue the phenotypes observed in Nanos2-null mice (Suzuki et al. 2012). Second, deletion of the NIM in *X. laevis* Nanos1 suppresses the abnormal development caused by the ectopic expression of wild-type Nanos1 in oocytes (Lai et al. 2011). Finally, *D. melanogaster* CCR4 (encoded by the *twin* gene) acts together with Nanos (Nos) and Pumilio (Pum), and *twin* mutants phenocopy the *nos* and *pum* mutant phenotypes in *D. melanogaster* ovarian germline stem cells (Joly et al. 2013).

### Direct recruitment of the CCR4–NOT complex by RNA-binding proteins

Our structural and functional studies provide a detailed molecular model for the recruitment of the CCR4–NOT complex by the NIM of Nanos proteins. Many other proteins exert their function by recruiting the CCR4–NOT complex to their mRNA targets, thereby repressing translation and/or promoting mRNA degradation. Prominent examples include the GW182 proteins, which are involved in miRNA-mediated gene silencing in animals; the *D. melanogaster* CUP and Smaug proteins; and TTP, a protein required for the degradation of mRNAs containing AU-rich elements (ARE-mediated mRNA decay) (Barckmann and Simonelig 2013). However, the available structural information on their mode of binding to the CCR4–NOT complex is limited. In addition to our structure, the only other structure that has been reported to date is of an N-terminal domain of CNOT1 bound to a short peptide of TTP (Fabian et al. 2013).

The comparison of the two protein families reveals similarities in their mode of action and CCR4–NOT recognition. TTP and Nanos proteins contain tandem zinc finger motifs, which are involved in RNA binding, and SLiMs embedded into extended disordered regions, which are required for the recruitment of the CCR4–NOT complex. The Nanos and TTP SLiM peptides form short helices that insert aromatic and hydrophobic residues into surface pockets between two α helices of the CNOT1 HEAT-like repeat domains (Fabian et al. 2013; this study). Furthermore, the overall shape and conformation of both peptides is stabilized by intramolecular hydrogen bonds. However, in contrast to the Nanos NIM, which binds to a very hydrophobic pocket in CNOT1 (Fig. 5B), the CNOT1–TTP interaction involves several polar contacts with the hydrophilic residues lining the binding pocket (Fabian et al. 2013). Another important difference is that the highly hydrophobic NIM-binding pocket of CNOT1 is masked in the apo structure and could not have been predicted in the absence of the ligand. Due to the hydrophobic nature of SLiMs, it is possible that other motifs may bind to their partners in similar hydrophobic pockets that are protected from solvent in their absence and undergo conformational changes upon binding.

In general, Nanos and TTP illustrate two mechanisms for how the CCR4–NOT complex can be recruited to specific mRNA targets. TTP combines sequence-specific RNA binding (ARE binding) with CNOT1 binding within one polypeptide chain, and Nanos requires Pumilio (or other partners) to specifically bind mRNA targets (Sonoda and Wharton 1999; Asaoka-Taguchi et al. 1999; Jaruzelska et al. 2003; Lai et al. 2011). This modular recruitment mode likely enhances the opportunities for regulation and confers redundancy and robustness to the repressive mechanism because Pumilio can also recruit the CCR4–NOT complex (Goldstrohm et al. 2006; Van Etten et al. 2012).

### Recruitment of the CCR4–NOT complex represents a common and widespread post-transcriptional regulatory mechanism

Our observation that the NIM peptides fused to a heterologous protein (i.e., GFP) can trigger both translational repression in the absence of mRNA decay and the degradation of bound mRNAs indicates that the recruitment of the CCR4–NOT complex to an mRNA target is sufficient to elicit these effects. Accordingly, previous studies have shown that, in addition to catalyzing deadenylation, the CCR4–NOT complex can repress translation independently of deadenylation (Cooke et al. 2010; Chekulaeva et al. 2011; Bawankar et al. 2013; Zekri et al. 2013).

The mechanism through which the CCR4–NOT complex triggers deadenylation and subsequent mRNA degradation is relatively well understood and involves deadenylation by the catalytic module, decapping through the recruitment of decapping complexes, and 5′-to-3′ mRNA degradation catalyzed by XRN1 (Wahle and Winkler 2013). However, little is known regarding how CCR4–NOT represses translation in the absence of mRNA deadenylation and decay. Given the growing number of RNA-binding proteins that recruit the CCR4–NOT complex to specific mRNAs, an important question for future studies is to determine the mechanisms through which this complex represses translation.

In summary, the CCR4–NOT complex is emerging as a major effector of translational repression and mRNA decay, funneling the effect of diverse post-transcriptional mRNA regulators into a common repressive pathway. The investigation of this repressive pathway will greatly enhance our understanding of post-transcriptional mRNA regulation in eukaryotic cells.

## Materials and methods

The DNA constructs are described in detail in the Supplemental Material and are listed in Supplemental Table 1. The antibodies used in this study are listed in Supplemental Table 2.

### mRNA reporter assays

For the tethering assays, HEK293T cells were seeded in six-well plates (0.6 × 10^6^ cells per well) and transfected using Lipofectamine 2000 (Invitrogen) or Turbofect (Thermo Scientific). The MS2-tethering reporters have been described previously (Lykke-Andersen et al. 2000). The transfection mixtures contained 0.5 μg of the control plasmid containing the β-globin gene fused to the GAPDH 3′ UTR but lacking MS2-binding sites (control; β-globin-GAP), 0.5 μg of the β-globin reporter containing six MS2-binding sites (β-globin-6xMS2bs), and various amounts of pCN-MS2-HA plasmids for the expression of MS2-HA fusion proteins. The cells were harvested 2 d after transfection. The total RNA was isolated using the Trifast reagent (Peqlab) and analyzed by Northern blot. For the tethering assays using the MALAT1 reporter, the transfection mixtures contained 0.5 μg of the control plasmid (pcDNA-F-Luc-V5) and 0.5 μg of pcDNA3-R-Luc-V5-6xMS2bs-MALAT1. When the R-Luc-Asb9 reporter was used, the transfection mixtures contained 0.05 μg of the R-Luc-Asb9 3′ UTR reporter plasmid, 0.1 μg of the pEGFP-N3-F-Luc plasmid, and 0.5 μg of the plasmids expressing MS2-HA-tagged Nanos proteins. The luciferase activity was measured using the dual-luciferase reporter assay system (Promega). To measure mRNA half-lives, transfected cells were treated with 10 μg/mL actinomycin D (final concentration) 2 d after transfection and harvested at the indicated time points. RNA samples were analyzed as described above. RNase H (New England Biolabs) digestion using a (dT)_15_ oligonucleotide was performed according to the manufacturer’s instructions.

### Protein expression, purification, and pull-down assays

All recombinant proteins were expressed in *E. coli* BL21 (DE3) Star cells (Invitrogen) in ZY medium for 5 h at 30°C or overnight at 20°C. The *Homo sapiens* CNOT1 SHD (residues 1833–2361) was expressed with an N-terminal MBP tag cleavable by the HRV3C protease. The cells were lysed in binding buffer containing 50 mM HEPES (pH 7.5), 300 mM NaCl, and 2 mM DTT supplemented with protease inhibitors, 1 mg/mL lysozyme, and 5 μg/mL DNase I. After purification using amylose resin (New England Biolabs) the protein was diluted in binding buffer containing 75 mM NaCl and further purified on a HiTrapQ ion exchange column (GE Healthcare) using a linear gradient to 1 M NaCl. For the GST pull-down assays, the MBP-CNOT1 SHD was purified using a Superdex 200 column (GE Healthcare) in gel filtration buffer containing 10 mM HEPES (pH 7.5), 300 mM NaCl, and 2 mM DTT. For crystallization, the MBP-CNOT1 protein eluted from the HiTrap-Q column was digested overnight with recombinant HRV3C protease purified from *E. coli*. The GST-tagged HRV3C protease and MBP were removed using HiTrap GST and MBP columns (GE Healthcare), respectively. The remaining contaminants were removed by size exclusion chromatography in gel filtration buffer. The expression and purification of the Nanos1–3 NIM peptides and the NOT module and SBP and GST pull-down assays are described in the Supplemental Material.

### Crystallization, data collection, and structure determination

The *H. sapiens* Nanos1 NIM peptide (residues 40–56) was chemically synthesized by EMC Microcollections and solubilized in gel filtration buffer. The initial crystals of the CNOT1 SHD bound to the Nanos1 NIM peptide were obtained within 1 d by sitting-drop vapor diffusion at 22°C in drops containing 0.2 μL of the protein complex solution (3.0 mg/mL CNOT1, 0.09 mg/mL Nanos1 NIM peptide in gel filtration buffer) and 0.2 μL of the reservoir solution (0.8 M succinic acid at pH 7.0). The optimized crystals that grew within 1 wk were obtained by microseeding onto hanging drops containing 1.0 μL of the protein complex solution (1.8 mg/mL CNOT1, 0.06 mg/mL Nanos1 in gel filtration buffer) and 0.6 μL of the reservoir solution (0.7 M succinic acid at pH 7.0). The crystals were cryoprotected using the reservoir solution supplemented with 20% glycerol and flash-frozen in liquid nitrogen.

A detailed description of the process used for data collection and structure determination can be found in the Supplemental Material. The refinement statistics are summarized in Table 1.

### Accession codes

The coordinates for the structure of CNOT1 SHD bound to the Nanos1 NIM peptide were deposited in the PDB under ID code 4CQO.
